# Black soldier fly (*Hermetia illucens*) larvae powder as a larval diet ingredient for mass-rearing *Aedes* mosquitoes

**DOI:** 10.1051/parasite/2019059

**Published:** 2019-09-19

**Authors:** Wadaka Mamai, Nanwintoum Sévérin Bimbilé Somda, Hamidou Maiga, Anna Konczal, Thomas Wallner, Mame Thierno Bakhoum, Hanano Yamada, Jérémy Bouyer

**Affiliations:** 1 Insect Pest Control Laboratory, Joint FAO/IAEA Division of Nuclear Techniques in Food and Agriculture PO Box 100 1400 Vienna Austria; 2 Institut de Recherche Agricole pour le Développement (IRAD) PO Box 2123 Yaoundé Cameroun; 3 Institut de Recherche en Sciences de la Santé/Direction Régionale de l’Ouest (IRSS/DRO) 01 PO Box 545 Bobo-Dioulasso Burkina Faso; 4 Laboratoire d’Entomologie Fondamentale et Appliquée (LEFA) Université Joseph Ki-Zerbo Ouagadougou 03 PO Box 7021 Burkina Faso

**Keywords:** Insect diets, Larval diets, Larval development, Quality control, Genetic control, Vectors, Arbovirus

## Abstract

The mass production of mosquitoes is becoming more wide-spread due to the increased application of the sterile insect technique (SIT) and other genetic control programmes. Due to the variable availability and high cost of the bovine liver powder (BLP) constituent of many current larval diets, there is an urgent demand for new ingredients in order to support sustainable and efficient mosquito production while reducing rearing cost, without affecting the quality of the insects produced. Two black soldier fly (BSF) powder-based diet formulations (50% tuna meal, 35% BSF powder, 15% brewer’s yeast and 50% tuna meal + 50% BSF powder) were tested for their suitability to support the development of *Aedes aegypti* and *Ae. albopictus* mosquitoes in mass-rearing conditions. Overall, the results indicate that the use of the BSF powder did not negatively impact the development and quality of the produced insects in terms of time to pupation, adult production and male flight ability. Furthermore, depending on the species and diet formulations, there were improvements in some parameters such as female body size, egg production, egg hatch rate and male longevity. BSF powder is a valuable ingredient that can effectively replace costly BLP for the mass production of high quality *Ae. aegypti* and *Ae. albopictus* mosquitoes. Both diet formulations can be used for *Ae. aegypti* showing high plasticity to nutrition sources. However, for *Ae. albopictus* we recommend the combination including brewer’s yeast.

## Abstract (fr)

L’élevage de masse de moustiques est de plus en plus répandu en raison de l’application de la technique de l’insecte stérile et d’autres techniques de lutte génétique. En raison de la disponibilité variable et du coût élevé de la poudre de foie de bovin, ingrédient de nombreux régimes larvaires, il devient urgent de trouver de nouveaux ingrédients afin de soutenir une production durable et efficace des moustiques, en réduisant les coûts d’élevage sans toutefois affecter la qualité des insectes produits. Deux formulations de régime à base de poudre de mouche-soldat noire (50 % farine de thon + 35 % poudre de mouche-soldat noire + 15 % levure de bière et 50 % farine de thon + 50 % poudre de mouche-soldat noire) ont été évaluées pour déterminer leur capacité à soutenir le développement larvaire d’*Aedes aegypti* et *Ae. albopictus* dans des conditions d’élevage de masse. Dans l’ensemble, les résultats indiquent que l’utilisation de la poudre de mouche-soldat noire n’a pas d’impact négatif sur le développement larvaire et la qualité des insectes produits en termes de temps de développement, de production d’adultes et de capacité de vol des mâles. En outre, en fonction de l’espèce et de la formulation du régime, certains paramètres tels que la taille des femelles, la production d’œufs, le taux d’éclosion des œufs et la longévité des mâles ont été améliorés. La poudre de mouche-soldat noire est un ingrédient de valeur qui peut remplacer efficacement la coûteuse poudre de foie de bovin pour la production en masse de moustiques *Ae aegypti* et *Ae. albopictus* de grande qualité. Les deux formules de régime peuvent être utilisées pour *Ae. aegypti* qui montre une grande plasticité à la source de nutrition. Cependant, pour *Ae. albopictus*, nous recommandons la formulation comprenant la levure de bière.

## Introduction

Some mosquito species play an important role in the transmission cycle of several human and animal diseases. *Aedes aegypti* and *Ae. albopictus* are very efficient vectors of arboviruses, including dengue, yellow fever, chikungunya and Zika. Traditional control methods, which largely rely on chemical insecticides, are not always effective due to the rapid spread of resistance of mosquitoes to these insecticides. Alternative, innovative mosquito control strategies such as the sterile insect technique (SIT) which involves the release of radio-sterilized insects, gene drive strategies, and *Wolbachia*-based strategies are additional, promising vector control tools that require the mass-rearing of mosquitoes and the continuous release of competitive adults in the target site over an extended period of time [11, 16, 23].

The SIT as a major component of area-wide integrated pest management (AW-IPM) has regained interest over the last few decades as a complementary tool to the current mosquito control methods to limit the transmission of mosquito-transmitted pathogens [1, 6, 7, 21, 28, 29, 40]. In the context of applying the SIT, larval rearing diet is one of the most critical and costly components of the operational running costs [22]. In order to develop the SIT package against mosquitoes at the Insect Pest Control Laboratory (IPCL) of the Joint FAO/IAEA Division of Nuclear Techniques in Food and Agriculture, efforts have been made toward the optimization of rearing methods at the larval stage, including development of equipment and protocols [2–4, 17, 31, 34–36, 38].

The aquatic phase is an important part of mosquito life. Larval nutrition is therefore a primary determinant of lifespan and has been shown to affect several life-history parameters in mosquitoes including larval development and resulting adult fitness. Therefore, it is important to balance high quality nutrition with production costs. A standard artificial larval diet consisting of tuna meal (TM), bovine liver powder (BLP), and brewer’s yeast (BY) has been developed and used successfully at the IPCL, Seibersdorf, Austria and in other laboratories for rearing *Aedes* and *Anopheles* mosquito species [10, 17, 24, 25, 41, 42]. Although this standard diet includes a variety of components such as sugars, fatty acids, proteins and vitamins [8, 17] necessary for larval growth and adult fitness, the principal protein source is BLP which is a common ingredient in many mosquito diets [8, 26, 49]. However, this is also the most expensive constituent and its widespread availability is not always guaranteed [25]. Since pilot field studies of SIT application against *Ae. aegypti* and *Ae. albopictus* are currently being undertaken in various countries around the world such as Italy, China and Brazil [6, 49], alternative, cost-effective, and readily available diet ingredients for mass-rearing *Aedes* sp. are urgently needed. Therefore, as in many other laboratories worldwide looking for a suitable and inexpensive diet [10], the IPCL has initiated activities to explore alternative sources of diet ingredients to replace BLP in an attempt to find a balance between diet effectiveness and cost [8, 9].

Insects are an attractive option for providing nutritional sources for rearing mosquitoes and reducing the environmental impact related to the production of protein from animal sources. They are rich sources of protein, essential amino acid and fat at all life stages [12, 46]. However, the nutrient concentration depends on their life stages, rearing conditions and the composition of the growth media used for insect production. The black soldier fly [*Hermetia illucens* (Linnaeus, 1758), Diptera: Stratiomyidae)], further referred to as “BSF”, a common and widespread fly, has been used as a model system for reducing waste and as feed for a variety of animals [5], including swine [39], poultry [15] and fish [43]. The proof of concept of rearing mosquitoes with flies including BSF has recently been demonstrated [9]. However, the use of BSF as an ingredient of mosquito larval diet has not been tested at a large scale. For any new ingredient, prior bioassay testing on a small scale should be performed to ensure its potential suitability. In this context, Bimbilé Somda et al. [9] have shown that the meal of *Tenebrio molitor* (Yellow mealworm), *Musca domestica* (House fly) and BSF can be used as a sustainable alternative to BLP to rear mosquitoes at a small scale. However, the house fly is a pest and potential disease vector. BSF is one of the more suitable insect candidates due to its short reproduction cycle, ease of rearing in large numbers, and the adults are neither pests nor vectors and rearing therefore requires no specific precautionary measures [5].

Following up on the previous evaluation [9], this study aimed to assess the use of BSF meal as a diet ingredient for mass-rearing *Ae. aegypti* and *Ae. albopictus.* Mosquito life-history parameters including larval development, production parameters, and the quality of the produced insects were assessed in comparison to the standard diet.

## Materials and methods

### Mosquito colonies and maintenance

Experiments were performed using two established mosquito colonies: *Ae. aegypti* originating from Juazeiro, Brazil since 2012 (provided by Biofabrica Moscamed, IAEA Collaborative Center) and *Ae. albopictus* originating from Rimini, Italy since 2018 (provided by Centro Agricoltura Ambiente, IAEA Collaborative Center), respectively. These strains were maintained in a 264 m^2^ refurbished container-based laboratory under controlled environmental conditions: the larval rearing room was maintained at 28 ± 2 °C, 80 ± 10% RH and the adult rearing room at 26 ± 2 °C, 60 ± 10% RH, with a 12:12 hour light: dark (L:D) photoperiod with 1 hour periods of simulated dawn and dusk in both rooms.

### Diet formulation and experimental design

The defatted and dry BSF larvae powder used in this study was purchased from InnovaFeed (Évry, France; http://www.innovafeed.com/), specialized in producing high quality insect meals for the feed industry or research and development purposes. The IAEA diet which consists of 50% TM + 35% BLP + 15% BY (hereafter diet A) [18] was used as a reference and control. Two BSF-based diets were selected based on the preliminary evaluation of Bimbilé Somda et al. [9]: (1) 50% TM + 15% BY + 35% BSF (hereafter diet B) and (2) 50% TM + 50% BSF (hereafter diet C). The diet ingredients were weighed individually with an electric balance, mixed together and finally diluted in deionized water to produce a 4% (w/v) suspension.

Mosquito eggs used in this study were obtained from mass-rearing cages following mass-rearing procedures developed at the IPCL [4, 18, 32]. Filter papers containing 2 week-old eggs were gently brushed off. Three sub-samples of 100–150 eggs were used to confirm the hatch rate of the particular egg batch. Based on the egg hatch rate, egg batches corresponding to ~18,000 first instars were estimated following the method described by Zheng et al. [48], weighed and then hatched separately in glass jam jars (IKEA of Sweden AB SE-343 81 Almhult, Germany) filled with 700 mL of boiled and cooled reverse osmosis water with the addition of 10 mL of the corresponding diet mixture (see [18, 32] for details). After hatching, the contents of jars (first-instar larvae) were sieved (50-μm sieve, Retsch® Test Sieve with steel mesh) and transferred into mass-rearing trays (L × W × H = 100 × 60 × 3 cm, Glimberger Kunststoffe GmbH., Austria) placed on a table and containing 5 L of reverse osmosis water (added 1 day before the addition of larvae to allow the water temperature to adjust to the ambient air temperature). Larvae were reared with 4% (w/v) larval diet of each diet mixture in the following amounts: 50 mL on day 1, 100 mL on day 2, 200 mL on day 3 and 4, 150 mL on day 5 and 50 mL from day 6 onwards. Four replicates of each diet formulation were performed.

Pupae were harvested on five consecutive days, from day 6 to 10 after hatching at 9 AM as follows: from each tray, larvae and pupae were sieved by using a 600-μm sieve (Retsch® Test Sieve with steel mesh) and transferred into small trays for sorting. The separation of larvae, male and female pupae were done mechanically using a Fay-Morlan [19] glass sorter as redesigned by Focks (John W. Hock Co., Gainesville, FL, USA) [20]. Larvae were returned to their corresponding trays with the same, used larval water. Larvae remaining after day 10 were discarded. The following mosquito life-history parameters were evaluated:

- time to pupation or larval development time, defined as the number of days between hatching and pupation calculated as the mean time that the larval stage lasted for each diet;
- pupation percentage (males and females) for each diet mixture, expressed as the percentage of larvae molted into pupae, calculated as the total number of pupae formed by the end of 5 days of pupae collection over the initial number of L1s;
- male adult production which is the total number of emerged males, in relation to the initial number of L1s;
- male emergence percentage: 100 pupae from each diet formulation and each day were placed into small bowls containing 50 mL of reverse osmosis water. These bowls were placed in individual cages (15 × 15 × 15 cm, BugDorm-1H, MegaView, Taichung, Taiwan). Dead pupae were counted to calculate the rate of emergence as a proportion of emerged adults from the total number of pupae;
- egg production and egg hatch percentage: 4000 pupae (3:1 ratio in favor of females) were transferred into 30 cm^3^ emergence cages (BugDorm, Taiwan) containing 10% sugar solution. At 4 days post emergence, females were offered a porcine blood meal on two consecutive days using collagen sausage casing (Grade Specification: 3, 26 NC, EDICAS Co., Ltd.) filled with 50 mL of blood. Eggs were collected from each cage by introducing oviposition cups lined with germination papers. The papers containing the eggs were removed from the cage after 4 days, air dried and stored for two weeks. Eggs were gently brushed off and their number estimated following the method described in Zheng et al. [48]. Three batches of eggs were evaluated. From each batch and replicate, 100–150 egg sub-samples were taken to determine egg hatch percentage;
- male flight ability: 120 male pupae from each rearing tray were separated from females under a stereomicroscope by distinguishing differences in genitalia [37]. After emergence was complete, the flight ability test was performed on 100 emerged adult male mosquitoes (4 day-old) following the protocol developed at the IPCL [14];
- male longevity: after the flight ability test, 50 males that had escaped and 30 mosquitoes that remained at the base of the flight tubes from each cage were removed separately using a mouth aspirator and transferred to a cage (15 × 15 × 15 cm, Bugdorm.com, Taiwan) for measurement of longevity. A 10% sugar solution was supplied in a 150-mL plastic cup containing a sponge. Mortality checks were carried out daily until no living adults remained;
- wing length: to determine whether diet treatment affected adult body size, 60 adults of each sex from each diet treatment were taken (15 samples were taken randomly from each replicate), their left wings dissected, and a photograph of each wing taken under a dissecting microscope (Leica, MZ16 FA, Leica Microsystems (Switzerland) Ltd.) for measurement. Wings were measured from the distal edge of the alula to the end of the radius vein (excluding fringe scales) [30] with analySIS FIVE software (Olympus Corporation, Tokyo, Japan).

## Statistical analysis

Statistical analyses were performed using R Software, version 3.5.2 (R Development Core Team 2008, URL http://www.R-project.org/) and GraphPad Prism v.5.0 ((Windows, Graphpad Software, La Jolla California, USA; www.graphpad.com). A Gaussian linear mixed-effects model was used with time to pupation, egg production, male and female body size assigned as response variables, diet mixture as a fixed effect and replicate as a random effect [27]. We also used binomial generalized linear mixed models fit by maximum likelihood (Laplace Approximation) with pupation, adult production, emergence, egg hatch percentages and flight ability as response variables, diet mixture as fixed effect, and the replicate as a random effect. The longevity of mosquitoes was analysed using Kaplan–Meier survival analyses. The log-rank (Mantel-Cox) test was used to compare the level of survival between different treatments. The Bonferroni correction method was applied for each pair of groups to account for the multiplicity comparisons.

## Results

The parameters assessed were selected according to their importance for a successful SIT programme. These include the fitness-related traits for males to be released (total male production, adult body size, longevity and flight ability), and fitness-related traits for females important for mosquito production in the facility (adult body size, egg production and egg hatch).

**Figure 1:**
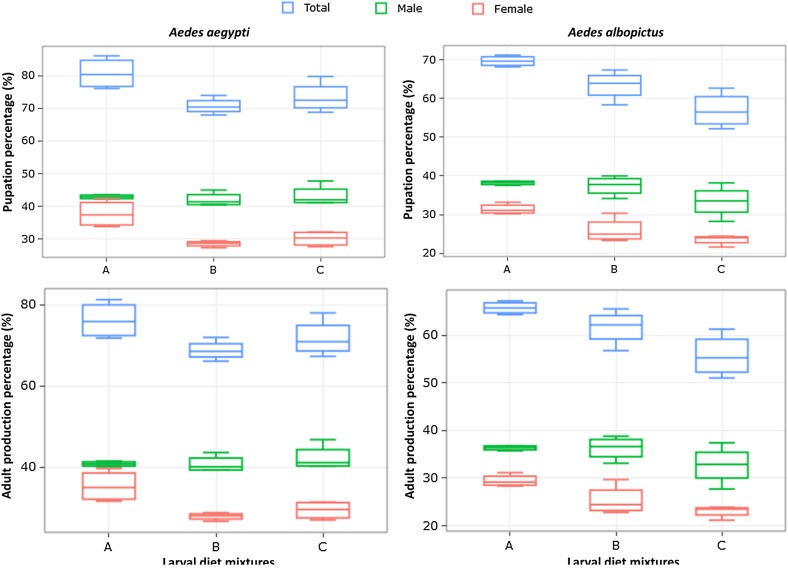
Pupation and adult production percentages in male and female *Aedes aegypti* and *Aedes albopictus.* Diet A = 50% TM + 35% BLP + 15% BY, Diet B = 50% TM + 35% BSF + 15% BY, Diet C = 50% TM + 50% BSF.

**Figure 2:**
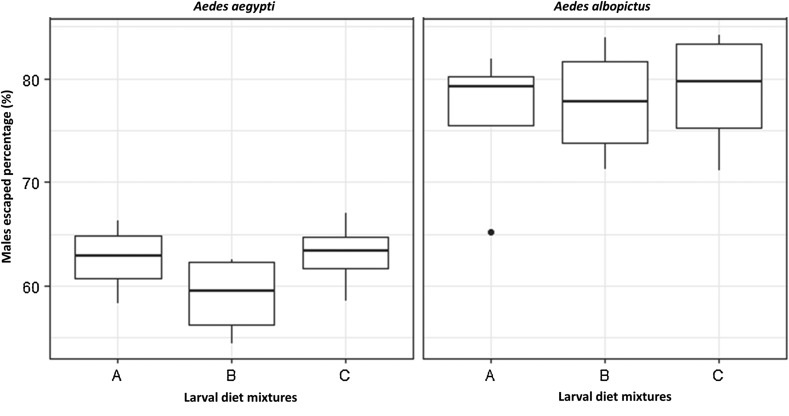
Flight ability of male *Aedes aegypti* and *Aedes albopictus.* Diet A = 50% TM + 35% BLP + 15% BY, Diet B = 50% TM + 35% BSF + 15% BY, Diet C = 50% TM + 50% BSF.

**Figure 3:**
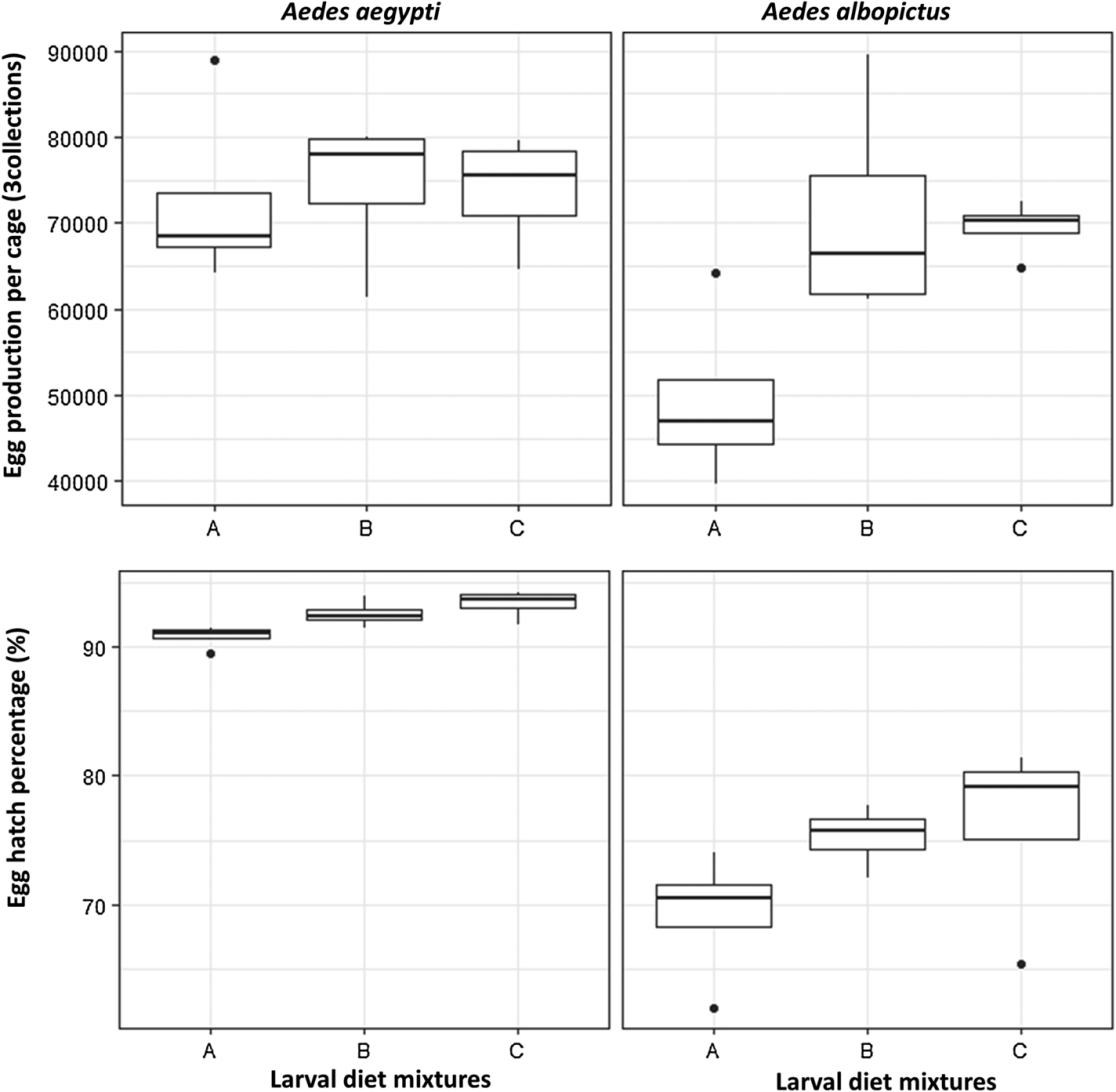
Egg production over three gonotrophic cycles and egg hatch rate in *Aedes aegypti* and *Aedes albopictus.* Diet A = 50% TM + 35% BLP + 15% BY, Diet B = 50% TM + 35% BSF + 15% BY, Diet C = 50% TM + 50% BSF.

**Figure 4:**
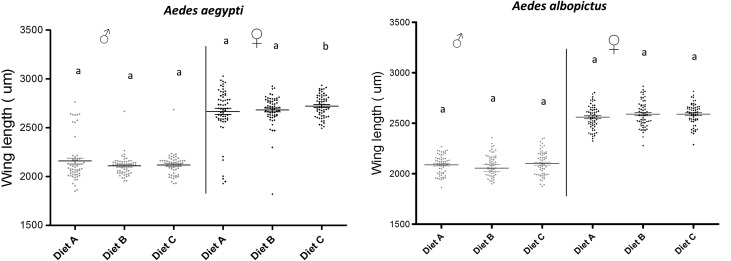
Wing length of male and female *Aedes aegypti* and *Aedes albopictus* reared from L1 with different larval diets. Different letters indicate significantly different results between treatments, by sex. Points represent individuals and the horizontal bar the mean. Diet A = 50% TM + 35% BLP + 15% BY, Diet B = 50% TM + 35% BSF + 15% BY, Diet C = 50% TM + 50% BSF.

**Figure 5:**
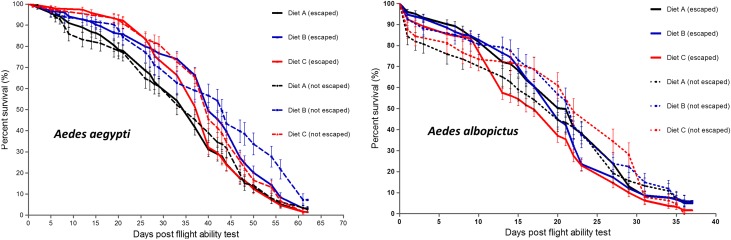
Longevity post flight ability test of male *Aedes aegypti* and *Aedes albopictus* reared from L1 with different larval diets. Diet A = 50% TM + 35% BLP + 15% BY, Diet B = 50% TM + 35% BSF + 15% BY, Diet C = 50% TM + 50% BSF.

Both *Aedes* species were successfully raised on BSF-based diets B and C until adult emergence. Results are summarised in Tables 1–3, Figures 1–5, and the statistical analyses in Tables 4 and 5.

**Table 1:** Time to pupation of *Aedes aegypti* and *Aedes albopictus* reared with different larval mixtures.

Species	Diet mixtures	Male time to pupation (days)	Female time to pupation (days)	Time to pupation (days)
*Aedes aegypti*	Diet A	7.55 ± 0.09	8.49 ± 0.05	7.99 ± 0.06^a^
	Diet B	7.48 ± 0.04	8.36 ± 0.15	7.84 ± 0.06^b^
	Diet C	7.54 ± 0.05	8.57 ± 0.07	7.96 ± 0.03^ab^
*Aedes albopictus*	Diet A	6.94 ± 0.04	7.87 ± 0.06	7.36 ± 0.04^a^
	Diet B	6.96 ± 0.07	7.94 ± 0.03	7.36 ± 0.04^a^
	Diet C	7.22 ± 0.04	8.13 ± 0.04	7.60 ± 0.03^c^

Different superscript letters indicate significant differences among diet treatments.

Time to pupation differed between diet mixtures and species (Table 1). In comparison with the reference diet A, the BSF-based diet B accelerated larval development in *Ae. aegypti* (*t* = −2.48, df = 6, *p* = 0.048) but not in *Ae. albopictus* (*t* = 0.12, df = 6, *p* = 0.91). Diet C did not affect the time to pupation in *Ae. aegypti* (*t* = −0.49, df = 6, *p* = 0.64) but increased it in *Ae. albopictus* (*t* = 5.41, df = 6, *p* = 0.002). Regardless of the species and the type of diet, mean time to pupation ranged from 7.25 to 8.10 days. Interestingly, *Ae. albopictus* had lower time to pupation than *Aedes aegypti* (Table 1). Delayed pupation of females in BSF-based diets was observed compared to insects reared on the standard diet (Table 1).

The percentages of male pupae varied between diet mixtures, ranging from 42.08 ± 1.28 to 43.23 ± 1.81% in *Ae. aegypti* and from 33.40 ± 2.35 to 38.19 ± 0.30 in *Ae. albopictus*. In females, these percentages ranged from 28.57 ± 0.52 to 37.75 ± 2.37 in *Aedes aegypti* and from 23.54 ± 0.73 to 31.43 ± 0.77 in *Aedes albopictus.* The percentages of female pupae were lower with diet B and C than with the standard diet in *Ae. aegypti* (Table 4, *p* < 0.05). More interestingly, the male emergence percentage when using BSF-based diets (B and C) was significantly higher in both species (Tables 4 and 5, Fig. 1). The percentage of adult production was significantly higher with diet C in *Ae. aegypti* (Table 4 and 5, Fig. 1, *z* = −5.82, *p* < 0.0001). In *Ae. albopictus*, similar percentages of male production were observed with diet B and reference diet A (*z* = −0.16, *p* = 0.87).

No significant variation in male flight ability (*p* > 0.05) occurred among the different diets in both species (Tables 2, 4, 5 and Fig. 2).

**Table 2:** Male pupation, emergence and adult production percentages, and flight ability of *Aedes aegypti* and *Aedes albopictus* reared with different larval mixtures.

Species	Diet mixtures	Male pupation rate (%)	Male emergence rate (%)	Male adult production (%)	Male flight ability (%)
*Aedes aegypti*	Diet A	42.92 ± 0.38^a^	95.12 ± 1.01^a^	40.82 ± 0.36^a^	62.61 ± 2.01^a^
	Diet B	42.08 ± 1.21^b^	96.94 ± 0.53^b^	40.80 ± 1.17^a^	59.00 ± 2.30^a^
	Diet C	43.23 ± 1.81^a^	97.93 ± 0.83^c^	42.33 ± 1.77^b^	63.06 ± 2.01^a^
*Aedes albopictus*	Diet A	38.19 ± 0.30^a^	97.85 ± 0.20^a^	36.33 ± 0.29^a^	76.40 ± 4.39^a^
	Diet B	37.43 ± 1.45^b^	99.25 ± 0.20^b^	36.29 ± 1.40^a^	77.67 ± 3.33^a^
	Diet C	33.40 ± 2.35^c^	98.25 ± 0.20^c^	32.71 ± 2.31^b^	78.71 ± 3.51^a^

TM = tuna meal, BLP = bovine liver powder, BSF = black soldier fly. Diet A = 50% TM + 35% BLP + 15% BY, Diet B = 50% TM + 35% BSF + 15% BY, Diet C = 50% TM + 50% BSF. Different superscript letters indicate significant differences among diet treatments. Values are Means ± SE.

The number of eggs laid per cage differed with diet mixture in *Ae. albopictus.* Using diets B and C resulted in significantly higher egg production in *Ae. albopictus* compared to reference diet A (Tables 3 and 5, Fig. 3, *z* = 4.61, df = 6, *p* = 0.004 and *z* = 4.31, df = 6, *p* = 0.005, for diet B and diet C, respectively). No significant difference in egg production was found between diet treatments in *Ae. aegypti*. However, diet C resulted in significantly higher egg hatch in *Ae. aegypti* (*z* = 3.52, df = 6, *p* = 0.01). *Aedes albopictus* egg hatch following BSF-based diets B and C was similar but significantly higher than that of reference diet A.

**Table 3:** Female pupation, fecundity, egg production and egg hatch rate of *Aedes aegypti* and *Aedes albopictus* reared with different larval mixtures.

Species	Diet mixtures	Female pupation (%)	Egg production/cage (three batches)	Egg hatch (%)
*Aedes aegypti*	Diet A	37.75 ± 2.37^a^	72,482 ± 6,472^a^	90.81 ± 0.52^a^
	Diet B	28.57 ± 0.52^b^	74,280 ± 5,069^a^	92.56 ± 0.77^a^
	Diet C	30.13 ± 1.31^c^	73,809 ± 3,910^a^	93.37 ± 0.73^b^
*Aedes albopictus*	Diet A	31.43 ± 0.77^a^	49,439 ± 6,026^a^	69.25 ± 3.57^a^
	Diet B	25.93 ± 1.81^b^	70,865 ± 7,629^b^	75.25 ± 3.23^b^
	Diet C	23.54 ± 0.73^c^	69,459 ± 1,912^b^	76.25 ± 3.95^b^

TM = tuna meal, BLP = bovine liver powder, BSF = black soldier fly. Diet A = 50% TM + 35% BLP + 15% BY, Diet B = 50% TM + 35% BSF + 15% BY, Diet C = 50% TM + 50% BSF. Different superscript letters indicate significant differences among diet treatments. Values are Means ± SE

The wing length measurements for males and females of both species reared with the three larval diets are shown in Figure 4. Rearing larvae of *Ae. aegypti* and *Ae. albopictus* with BSF-based diet (diets B and C) did not negatively impact the wing length (Tables 4 and 5). More importantly, diet C significantly increased the wing length of female *Ae. aegypti* (*t* = 2.24, df = 351, *p* = 0.03). As expected, females of both *Aedes* species and from different diet treatments were significantly larger than their male counterparts.

**Table 4:** Results of the linear mixed model and binomial generalized linear mixed model for the effect of diet mixtures on *Aedes aegypti* life history trait parameters. Values were compared to reference diet A.

Species	Parameters		Value	SE	DF	*t*-value	*p*-value
*Aedes aegypti*	Time to pupation	Intercept	7.36	0.04	6	181.33	**<0.0001**
		Diet B	0.005	0.06	6	−2.48	**0.048**
		Diet C	0.24	0.06	6	−0.49	0.64
	Male body size	Intercept	2087.99	21.18	351	101.93	**<0.0001**
		Diet B	−33.19	29.95	351	−1.66	0.098
		Diet C	12.21	29.95	351	−1.94	0.05
	Female body size	Intercept	2558.57	24.37	351	109.35	**<0.0001**
		Diet B	30.18	27.72	351	0.58	0.56
		Diet C	29.70	27.72	351	2.24	**0.026**
	Egg production	Intercept	49,439.70	4551.89	6	15.92	**<0.0001**
		Diet B	21,426.14	3815.22	6	0.47	0.65
		Diet C	20,019.89	3815.22	6	0.35	0.74
			Estimate	SE		*z*-value	*p*-value
	Male pupation (%)	Intercept	−0.48	0.03		−8.80	**<2e-16**
		Diet B	−0.03	0.01		−3.20	**0.001**
		Diet C	−0.21	0.01		1.18	0.24
	Male adult production (%)	Intercept	−0.56	0.03		−11.66	**<2e-16**
		Diet B	−0.001	0.01		−0.11	0.91
		Diet C	−0.16	0.01		5.82	**6.08e-09**
	Male emergence (%)	Intercept	2.97	0.03		112.48	**<2e-16**
		Diet B	0.49	0.04		11.38	**<2e-16**
		Diet C	0.89	0.05		18.59	**<2e-16**
	Female pupation (%)	Intercept	−0.78	0.04		−11.16	**<2e-16**
		Diet B	−0.27	0.01		−36.94	**<2e-16**
		Diet C	−0.40	0.01		−30.52	**<2e-16**
	Male flight ability (%)	Intercept	0.51	0.11		4.67	**2.98e-06**
		Diet B	−0.20	0.16		−1.23	0.22
		Diet C	0.02	0.15		0.16	0.87
	Egg hatch (%)	Intercept	2.29	0.09		26.47	**<2e-16**
		Diet B	0.23	0.13		2.68	0.07
		Diet C	0.35	0.13		0.90	**0.007**

SE = standard error, DF = degree of freedom. Diet A = 50% TM + 35% BLP + 15% BY, Diet B = 50% TM + 35% BSF + 15% BY, Diet C = 50% TM + 50% BSF. Bold values are statistically significant.

**Table 5:** Results of the linear mixed model and binomial generalized linear mixed model for the effect of diet mixtures on *Aedes albopictus* life history trait parameters. Values were compared to reference diet A.

Species	Parameters		Value	SE	DF	*t*-value	*p*-value
*Aedes albopictus*	Time to pupation	Intercept	7.36	0.03	6	217.64	**<0.0001**
		Diet B	0.005	0.04	6	0.12	0.91
		Diet C	0.24	0.04	6	5.41	**0.002**
	Male body size	Intercept	2087.99	21.18	351	98.60	**<0.0001**
		Diet B	−33.19	29.95	351	−1.11	0.27
		Diet C	12.21	29.95	351	0.41	0.68
	Female body size	Intercept	2558.57	24.37	351	104.97	**<0.0001**
		Diet B	30.18	27.72	351	1.09	0.28
		Diet C	29.70	27.72	351	1.07	0.28
	Egg production	Intercept	49,439.70	4954.45	6	9.98	**0.0001**
		Diet B	21,426.14	4647.20	6	4.61	**0.004**
		Diet C	20,019.89	4647.20	6	4.31	**0.005**
			Estimate	SE		*z*-value	*p*-value
	Male pupation (%)	Intercept	−0.48	0.03		−15.37	**<2e-16**
		Diet B	−0.03	0.01		−2.98	**0.003**
		Diet C	−0.21	0.01		−18.97	**<2e-16**
	Male adult production (%)	Intercept	−0.56	0.03		−18.10	**<2e-16**
		Diet B	−0.001	0.01		−0.16	0.87
		Diet C	−0.16	0.01		−14.44	**<2e-16**
	Male emergence (%)	Intercept	2.97	0.03		106.11	**<2e-16**
		Diet B	0.49	0.05		10.79	**<2e-16**
		Diet C	0.89	0.05		16.73	**<2e-16**
	Female pupation (%)	Intercept	−0.78	0.02		−35.78	**<2e-16**
		Diet B	−0.27	0.01		−23.06	**<2e-16**
		Diet C	−0.40	0.01		−33.44	**<2e-16**
	Male flight ability (%)	Intercept	1.20	0.15		7.84	**4.66e-15**
		Diet B	0.08	0.18		0.47	0.64
		Diet C	0.13	0.18		0.72	0.47
	Egg hatch (%)	Intercept	0.81	0.07		12.34	**<2e-16**
		Diet B	0.30	0.09		3.28	**0.001**
		Diet C	0.35	0.09		3.84	**0.0001**

SE = standard error, DF = degree of freedom. Diet A = 50% TM + 35% BLP + 15% BY, Diet B = 50% TM + 35% BSF + 15% BY, Diet C = 50% TM + 50% BSF. Bold values are statistically significant.

Longevity of males taken from the flight test is summarized in Figure 5. Overall, there was a significant variation in longevity between treatments in *Ae. aegypti* (Log-rank (Mantel-Cox) test, *χ* ^2^ = 23.73, df = 5, *p* < 0.0002) and *Ae. albopictus* (Log-rank (Mantel-Cox) test, *χ* ^2^ = 13.35, df = 5, *p* = 0.02). Rearing larvae with diet B significantly increased the longevity of adult males in *Ae. aegypti* (Fig. 5, Log-rank (Mantel-Cox) test, *χ* ^2^ = 11.09, df = 1, *p* = 0.001) and diet C decreased the longevity of adult males in *Ae. albopictus* (Fig. 2, Log-rank (Mantel-Cox) test, *χ* ^2^ = 1.302, df = 1, *p* < 0.0001). Overall, in both species, there were no significant differences between males that escaped and those that did not (Log-rank (Mantel-Cox) test, *χ* ^2^ = 4.95, df = 1, *p* = 0.03 and Log-rank (Mantel-Cox) test, *χ* ^2^ = 5.34, df = 1, *p* = 0.02) for *Ae. aegypti* and *Ae. albopictus,* respectively.

## Discussion

For a successful SIT programme, it is important that the larval diet leads to high rearing productivity and subsequent quality of the adults produced. Although the BLP in the IAEA standard diet worked well, this is a costly ingredient and difficult to procure to many FAO and IAEA member states. Although the use of insects to feed insects has received little attention in the past, it is becoming attractive as an alternative ingredient and as a way to reduce the rearing cost. As the rearing duration can affect production costs [24], it is important that the diet supports the reduction of larval development time. Our data showed that the BSF-based diet, namely diet B, significantly decreased the larval developmental time in *Ae. aegypti*, while this remained similar to the standard diet in *Ae. albopictus.* These results are in line with the previous results obtained by Bimbilé Somda et al. [9] who found that *Ae. aegypti* and *Ae. albopictus* larvae developed to adulthood being fed only with pure BSF meal in normal rearing conditions. Most of the diets developed for rearing *Ae. aegypti* such as the laboratory rodent diet used at the Centro Regional de Investigacion en Salud Publica, Tapachula, Mexico [10] and for *Ae. albopictus* at the Centro Agricultura Ambiente, Italy using cat food, brewer’s yeast and tetramin fish food [41] resulted in longer times to pupation compared with the IAEA diet. In this regard, the BSF-based diets present an advantage due to their shorter times to pupation. However, the difference between the two BSF-based diets may only rely on the quantity of nutrients, as their proportions are different.

Producing a large number of males of high quality is a core requirement for the success of the SIT. We found that diet B did not negatively affect male adult production percentage in both species and even increased male production in *Ae. aegypti*. However, diet C substantially decreased male production in *Ae. albopictus*. Although pupation percentages with BSF diets were lower than with the reference diet, adult male production percentages were compensated by a significantly higher percentage of successful mosquito emergence. Interestingly, the quality of adults produced with larvae fed on BSF-based diets was not negatively affected. The flight ability of males reared with BSF-based diets was comparable to the reference diet in both species, indicating that the BSF-based diets might provide a nearly optimal balance of nutrients like the standard diet. Although the larval development time in females was slightly delayed in BSF-based diets, results showed that there was no negative effect or even increased body size of the females produced. The differential development time in males and females when fed on BSF-based diets could likely lead to increased size dimorphism between male and female pupae, which is an important determinant for sex separation and could also be exploited to obtain high male proportions for the first pupal sort as shown in Zhang et al. [47], which is beneficial in the SIT applications. More interestingly, there was an increase in female body size and egg hatch in *Ae. aegypti* and in egg production in *Ae. albopictus*. Body size reflects nutritional status and the level of teneral reserves in female mosquitoes is known to affect their body size, fecundity, longevity and blood meal consumption and utilisation [13, 38]. It has been shown that adult teneral reserves play a role in the pre-vitellogenic phase of ovarian development in anautogenous mosquitoes [45]; females use reserves carried over from the larval stage to develop the first batch of eggs. Poor larval nutrition reduces the body size and therefore fecundity. In general, the large females are more likely to ingest more blood and lay more eggs than small ones [44]. Our study showed that the average number of eggs laid per cage over three gonotrophic cycles by *Ae. albopictus* reared with BSF-based diets was significantly higher compared to the standard diet. Furthermore, male *Ae. aegypti* showed a moderate, but statistically significant increase in lifespan when fed on diet B. A larval diet for mosquitoes must provide sufficient and balanced amounts of nutrients to successfully support not only larval growth but also the production of adults of high quality. BSF contains high amounts of proteins, fats and calcium [5, 15] although their concentration can vary with life stage, rearing conditions and diet processing [33]. Therefore, this effectiveness of BSF-based diets may be attributed to its high nutritional value. Bimbilé Somda et al. [9] have found BSF comparable to BLP in terms of composition in crude proteins, sugar and polyunsaturated fatty acids. However, it is not clear how variations in each nutrient influence allocation to various mosquito functions. In this study, diet treatment did not affect the ability of mosquitoes to escape the flight tubes. It is expected or assumed that mosquitoes that escaped should be of greater quality and hence of greater longevity. Surprisingly, we found that mosquitoes that did not escape the flight tubes survived as long as those that escaped, suggesting that flight ability might not necessarily be correlated to longevity. Since longevity was measured after the flight test, it is possible that escaping may have played a role in consuming a certain amount of their energy compared to immobile mosquitoes. The increased longevity observed in mosquitoes that did not escape when fed with diet C further supports this hypothesis. However, this deserves further studies to elucidate the relation between flight ability and longevity.

BLP accounts for 92.1% of the cost of the standard diet [9]. When substituted with BSF, which costs approximately 8.5 euros per kilogram according to the manufacturer, this results in major economic savings of approximately 80% compared to the standard diet. The BSF-based diet B presented in this study has been used successfully at the IPCL for at least five generations in mass-rearing conditions and at a small scale for colony maintenance, without any adverse effects observed. However, further investigations are needed to determine whether BSF-based diets are capable of maintaining vital biological parameters for insect survival, reproduction and normal behaviour for several generations.

## Conclusion

The use of defatted BSF larva powder as a larval diet ingredient resulted in comparable production and improved quality of the mass-reared *Ae. albopictus* and *Ae. aegypti*, in comparison to the reference IAEA diet. Although both BSF-based diet formulations can be used for *Ae. aegypti,* we recommend the combination comprising brewer’s yeast for mass-rearing *Aedes* species. This evaluation has important implications in the design and planning of operational SIT activities.

## Data Availability

All data generated or analysed during this study are included in this published article.
